# Antimicrobial photodynamic therapy suppresses dental plaque formation in healthy adults: a randomized controlled clinical trial

**DOI:** 10.1186/1472-6831-14-152

**Published:** 2014-12-15

**Authors:** Akiko Ichinose-Tsuno, Akira Aoki, Yasuo Takeuchi, Teruo Kirikae, Takuro Shimbo, Masaichi-Chang-il Lee, Fumihiko Yoshino, Yutaka Maruoka, Toshiyuki Itoh, Isao Ishikawa, Yuichi Izumi

**Affiliations:** Division of Oral Maxillofacial Surgery, National Center for Global Health and Medicine, Shinjuku-ku, Tokyo, Japan; Department of Periodontology, Graduate school of Medical and Dental Sciences, Tokyo Medical and Dental University, 1-5-45 Yushima, Bunkyo-Ku, Tokyo, 113-8549 Japan; Department of Infectious Diseases, Research Institute, National Center for Global Health and Medicine, Shinjuku-ku, Tokyo, Japan; Ohta Nishinouchi Hospital, Koriyama, Fukushima, Japan; Yokosuka-Shonan Disaster Health Emergency Research Center & ESR Laboratories, Kanagawa Dental University Graduate School, Yokosuka, Kanagawa, Japan; Department of Oral Science, Kanagawa Dental University Graduate School, Yokosuka, Kanagawa, Japan; Department of Clinical Research, Center for Clinical Sciences, National Center for Global Health and Medicine, Shinjuku-ku, Tokyo, Japan; Institute of Advanced Biomedical Engineering and Science, Tokyo Women’s Medical University, Shinjuku-ku, Tokyo, Japan

**Keywords:** Antimicrobial photodynamic therapy, Red light emitting diode, Toluidine blue O, Dental plaque control, Hydroxyl radical, Randomized controlled trial

## Abstract

**Background:**

Oral care is important for oral and systemic health, especially for elderly institutionalized individuals and compromised patients. However, conventional mechanical plaque control is often difficult for these patients because of the pain or the risk of aspiration. Although antimicrobial photodynamic therapy (aPDT), which is considered an alternative or adjunct to mechanical approaches, has potential application as a less stressful method of daily plaque control, no clinical application of this technique has been reported.

**Methods:**

We investigated the inhibitory effect of a combination of toluidine blue O (TBO), and a red light-emitting diode (LED) on dental plaque formation in healthy volunteers. The optimal concentration of TBO was determined in preliminary *in vitro* experiments to evaluate the bactericidal effect of aPDT on *Streptococcus oralis* and to clarify its safety in fibroblast cells. To survey the mechanism of TBO-mediated aPDT, the quality and quantity of reactive oxygen species (ROS) generated during aPDT were also examined using electron spin resonance (ESR) spectroscopy. Subsequently, the inhibitory effect of aPDT on dental plaque formation was investigated in eleven subjects as a clinical pilot study. The right or left mandibular premolars were randomly assigned to the treatment (with aPDT) or control (without aPDT) groups. In total, aPDT was applied six times (twice per day) to the teeth in the test group over a period of four days. On the fourth day, the study concluded and the analyses were performed.

**Results:**

A combination of 500 or 1000 μg/ml TBO and LED irradiation for 20 s significantly decreased the number of colony forming units of *Streptococcus oralis*. The cytotoxicity of aPDT was comparable to that of standard antiseptics used in the oral cavity. Hydroxyl radicals were detected by ESR analysis, but singlet oxygen was not. A randomized controlled trial demonstrated that aPDT with 1000 μg/ml TBO and red LED irradiation significantly suppressed dental plaque formation without harming teeth or the surrounding tissues.

**Conclusions:**

aPDT has the potential to be a promising novel technical modality for dental plaque control.

**Trial registration:**

This trial was registered with University Hospital Medical Information Network Clinical Trials Registry (number UMIN000012504).

**Electronic supplementary material:**

The online version of this article (doi:10.1186/1472-6831-14-152) contains supplementary material, which is available to authorized users.

## Background

Antimicrobial photodynamic therapy (aPDT) is a procedure in which oxygen-dependent activation of a photosensitizer by light (mainly lasers) leads to the generation of cytotoxic reactive oxygen species (ROS). Recently, this procedure was introduced in the medical [1, 2] and dental [3–6] fields. Besides confirmation of its antimicrobial potential *in vitro* [7, 8], prior clinical studies have evaluated the application of aPDT for the treatment of acne vulgaris in dermatology [9], and of periodontal [10, 11], endodontic [12, 13], and peri-implant [14] disease in dentistry. Further, since it uses light-generated compounds, aPDT may be able to eradicate multidrug-resistant bacteria without influencing the emergence of further resistance in those bacteria [15].

Increasing evidence exists that oral care is critical for systemic health, especially in compromised patients. Previously, Yoneyama *et al*. reported that good oral care lowers the risk of pneumonia and the rate of mortality in elderly institutionalized individuals [16, 17]. Abe *et al*. also suggested that oral hygiene was effective in the prevention of influenza [18]. The US National Institutes of Health (NIH) has recommended that “all cancer patients should have an oral examination before the initiation of cancer therapy, and the treatment of preexisting or concomitant oral disease is essential in minimizing oral complications in all cancer patients” [19]. Conventionally, mechanical tools such as a toothbrush, dental floss, or sponge brush accomplish the removal of dental bacterial plaque. However, mechanical plaque control is technically demanding, and can be physically stressful for subjects with a decreased range of arm motion, or a medical condition that impedes them from maintaining good oral hygiene. Therefore, the application of a non-mechanical means for controlling dental plaque formation could be of wide interest.

In 1993, Wilson *et al*. [20] proposed aPDT as an alternative to pharmaceutical and mechanical means of eliminating dental bacterial plaque. However, there are no clinical reports on the use of aPDT as a preventive oral care method for the control of dental plaque formation. If aPDT could be used as a novel method to control dental plaque formation, patients may be able to receive efficient oral care without the unpleasant stresses that accompany conventional mechanical tooth cleaning, such as bleeding or pain.

Accordingly, we aimed to investigate the inhibitory effects of aPDT in the oral cavity of healthy volunteers as a pilot study, prior to the clinical trial involving actual patients. We focused on toluidine blue O (TBO), which is a classic photosensitizer of phenothiazinium salt, because its bactericidal effects were already clarified in previous *in vitro* studies [8, 21–23], as well as in the treatment of periodontitis [4]. Further, the bactericidal effects of TBO-mediated aPDT using high-power red light-emitting diode (LED) on two typical periodontopathic bacteria, *Porphyromonas gingivalis* and *Aggregatibacter actinomycetemcomitans*, have also been demonstrated *in vitro* [3].

Before the pilot study in healthy volunteers, the antimicrobial effects of aPDT on *Streptococcus oralis (S. oralis),* one of the typical facultative anaerobic bacterium in human dental plaque, and the cytotoxic effect of aPDT on fibroblasts, were examined *in vitro*. Further, the characterization of ROS generated during aPDT treatment was investigated by electron spin resonance (ESR).

## Methods

### Experiment 1: *In vitro*evaluation of bactericidal effects of aPDT on *Streptococcus oralis*

#### Preparation of bacterial planktonic suspension

*S. oralis* OMZ 607 was maintained on blood agar plates (E-MP23; Eiken Chemical Co. Ltd., Tochigi, Japan) at 37°C under aerobic conditions. A loopful of each strain was inoculated in 9 ml brain heart infusion (BHI) broth, and cultured anaerobically at 37°C for 16 h. Afterwards, 500 μl of the bacterial cell suspension was transferred into 5 ml of fresh BHI broth, and further incubated anaerobically at 37°C for approximately 5 h. Finally, a bacterial suspension of 10^8^ cells/ml was prepared using a counting chamber, and stored on ice until use.

#### Photosensitizer and light source

Toluidine blue O (TBO) powder (maximum absorption = 626 nm, Sigma, St. Louis, MO) was dissolved at concentrations of 100, 500, and 1000 μg/ml in sterile saline solution. A prototype high-power red LED device (active elements = AlInGaP, wavelength = 600–700 nm, peak wavelength = 660 nm, power density = 1.1 W/cm^2^, spot size = 9 mm at the device end; modified from Pencure™ with a 660 nm band Deep Red LED [LZ1-00R205; LedEngin, Inc., Santa Clara, CA] by J Morita Mfg. Kyoto, Japan) was used as the light source. The irradiation time of LED was fixed at 20 s, according to the results of our previous *in vitro* study [3], which demonstrated effective bacterial elimination using the TBO-mediated aPDT procedure with 20 s irradiation.

#### Lethal photosensitization

A 30-μl aliquot of bacterial suspension was mixed with saline solution or an equal volume of TBO solution at the various concentrations (100, 500, and 1000 μg/ml) in the wells of a sterile 96-well flat bottom plate (Falcon®; Becton Dickinson Co., NJ). The final concentrations of TBO in the mixed solution were 50, 250, and 500 μg/ml, respectively. After incubation at room temperature for 20 s, LED irradiation was performed for 20 s. The light-emitting end (diameter = 8 mm) of the LED was positioned to correspond with the opening of the well (diameter = 7 mm) during irradiation. The distance between the top surface of the mixed bacterial suspension and the light-emitting end was 7 mm, and the depth of the mixed solution was 3 mm. The actual power at the bacterial suspension surface was 310 mW, and the power density was calculated to be 0.94 W/cm^2^ (total energy 6.2 J for 20-s irradiation). Each bacterial suspension was individually exposed to LED irradiation after preparation of the suspension in each well. A total of 7 experimental groups (exposure to 100, 500, 1000 μg/ml TBO only, combination of TBO and 20 s LED irradiation, and 20 s LED irradiation only) and one untreated control group were prepared for each one well.

After treatment, a 10-μl aliquot from each well was serially diluted 10^2^–10^5^-fold with saline solution, and 10 μl of the diluted samples were plated in triplicate on blood agar plates. All of the procedures including solution preparation, irradiation, and plating samples were performed for each well individually (i.e. one by one). The 96-well plates were incubated aerobically at 37°C for 48 h, and the numbers of colony-forming units (CFUs) were determined. The experiment was repeated independently five times.

### Experiment 2: cytotoxic effect of aPDT on fibroblasts

#### Cell culture

Mouse fibroblast cell line L929 (Riken, Saitama, Japan) was cultured in 75 cm^3^ tissue culture flasks in 20 ml RPMI 1640 medium (Nacalai Tesque, Kyoto, Japan) containing 100 U/ml penicillin, 100 U/ml streptomycin, and supplemented with 2.5 mmol/l L-glutamine and heat-inactivated 5% fetal calf serum (Gibco®).

#### Cell treatment

1 × 10^4^ cells were seeded into each well of 96-well black assay plates (clear flat bottom; Costar®; Corning, NY), and incubated at 37°C in a humidified incubator with 5% CO_2_ for 48 h until the cell monolayer became confluent.

For experimental groups, after removal of the medium, 100-μl TBO (100, 500, or 1000 μg/ml) was added to each well. Cells were incubated for 20 s, and the TBO solution was then aspirated from all wells. After the cells were washed twice with 100-μl PBS, 100-μl medium was added to the well, and immediately the cell was exposed to LED light from the top of the plate at 0.94 W/cm^2^ for 20 s (total energy = 6.2 J). The controls were either untreated, or treated with TBO (100, 500, and 1000 μg/ml) only, and were washed twice with PBS.

The group of cells treated with 1000 μg/ml TBO and LED was compared with a group of cells treated with 2.5–3% H_2_O_2_ (Oxydol; Yoshida Pharmaceutical Co. Ltd., Saitama, Japan), or 0.025% benzalkonium chloride (OSVAN®; Nihon Pharmaceutical Co. Ltd., Tokyo, Japan), which are the standard antiseptics for oral mucosa. The cells were exposed to H_2_O_2_ and benzalkonium chloride for 20 s, and washed twice with PBS. All experiments were performed in triplicate and repeated five times.

#### Cytotoxicity assay

A colorimetric assay containing the tetrazolium compound 3-(4,5-dimethylthiazol-2-yl)-5-(3-carboxymethoxyphenyl)-2-(4-sulfophenyl)-2H-tetrazolium, inner salt; MTS (CellTiter 96® Aqueous One Solution Cell Proliferation Assay; Promega, WI), was used to determine cell viability [21]. About 100-μl aliquots of growth medium and 20 μl of MTS reagent were added to each well. Following incubation for 2 h at 37°C in a 5% CO_2_ incubator, the absorbance of each well was measured at 490 nm using a microplate reader, and cell viability was calculated.

### Experiment 3: analysis of ROSs generated during aPDT

ESR spectroscopy was used for the qualitative and quantitative evaluation of ROS generated during aPDT. The spin trap reagents 2,2,6,6-tetramethyl-4-piperidinol (4-OH-TEMP; 98% purity; Sigma), and 5,5-dimethyl-1-pyrroline-*N*-oxide (DMPO; Dojin Chemicals, Kumamoto, Japan) were used for singlet oxygen and hydroxyl radical detection, respectively. A mixture of 800-μl PBS, 100-μl 1000 μg/ml TBO, and 100-μl 4-OH-TEMP was used for measuring singlet oxygen. A mixture of 400-μl PBS, 50-μl 50 mM DMPO, and 50-μl of 1000 μg/ml TBO was used to measure hydroxyl radicals. Measurements were obtained after transfer of the mixtures to a quartz flat cuvette and exposure to the red LED light for 20 s. The measurement was performed using ESR (JES-RE 3X, JEOL; Tokyo, Japan) connected to a WIN-RAD ESR Data Analyzer (Radical Research, Tokyo, Japan) at the following instrument settings: microwave power = 8 mW, magnetic field = 335.8 mT, modulation width = 0.079 mT, sweep time = 1 min, and time constant = 0.03 s. All experiments were performed in triplicate at room temperature.

### Experiment 4: inhibition effect of aPDT on dental plaque formation

**Figure 1:**
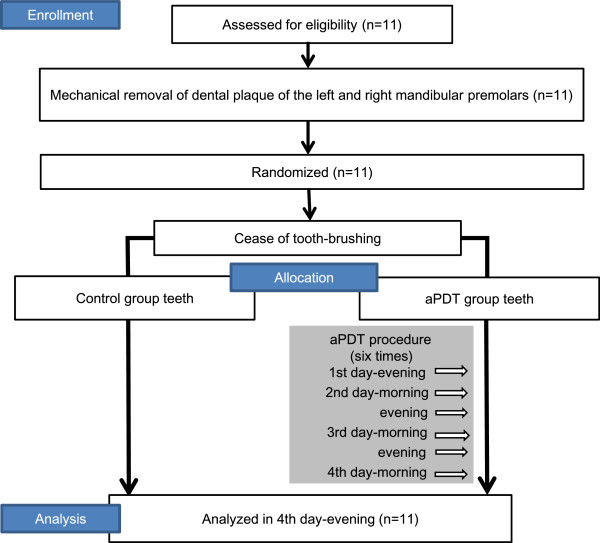
**Consort flow diagram of the clinical trial of the effectiveness of aPDT for inhibition of dental plaque.**

An open randomized single-blinded clinical trial with split-mouth design (each subject received test and control treatments, each to a separate side of the mouth) was conducted to investigate the inhibitory effect of aPDT on dental plaque formation (Figure 1).

#### Subjects

This clinical trial was approved by the Ethics Committee of the Faculty of Dentistry, Tokyo Medical and Dental University (TMDU) (No. 836), and was performed in accordance with the Declaration of Helsinki. This trial was registered with University Hospital Medical Information Network Clinical Trials Registry (number UMIN000012504) and was performed by following the CONSORT guidelines for clinical trials Additional file 1. The recruitment of subjects and experiment were conducted from January 2013 to February 2013 at Department of Periodontics, Dental Hospital of TMDU.

Eleven volunteer dentists were recruited for this study, and informed consent was obtained from all participants. The subjects included seven men and four women, aged 26–33 years (mean = 28.0 ± 2.3 years). Inclusion criteria for subject enrollment included good general health, no antimicrobial intake in the last three months, the presence of at least 20 teeth, the normal eruption of all mandibular premolars, the absence of swelling or redness of the gingiva, and the absence of sites with probing depth ≥ 4 mm.

#### Sample size calculation

The sample size was calculated using a statistical power of 80%, Type I error rate of 5%, and an assumed difference of 10% and SD of 10% with the values of plaque deposition area. On the basis of these data, the number of subject required to conduct this study was calculated as 10. However, considering the possibility of having one drop-out subject, enrollment of 11 participants was planned.

#### The aPDT procedure

The first and second premolars on the left and right sides of the mandible were evaluated. The selection of teeth was based on location, and suitability for LED irradiation, photographing, and sample collection, as well as plaque accumulation given the study conditions. The clinical trial was performed over four days, beginning and ending in the evening (Figure 1). On the first day, the dental plaque deposited on the buccal and lingual surfaces of the mandibular premolars on both sides was dyed red with a plaque disclosing solution (PROSPEC®; GC, Tokyo, Japan). The supra- and sub-gingival plaque was then thoroughly removed by professional tooth cleaning (PTC) using an ultrasonic scaler, as well as a rubber cup and a cone-shape brush mounted on a micromotor handpiece.

Subsequently, the right or left side premolars were randomly assigned for treatment (with aPDT) or as non-treated controls (without aPDT) by using the envelope method, in which the participants randomly selected an opaque envelope containing a note allocating the treatment side (right or left).

**Figure 2:**
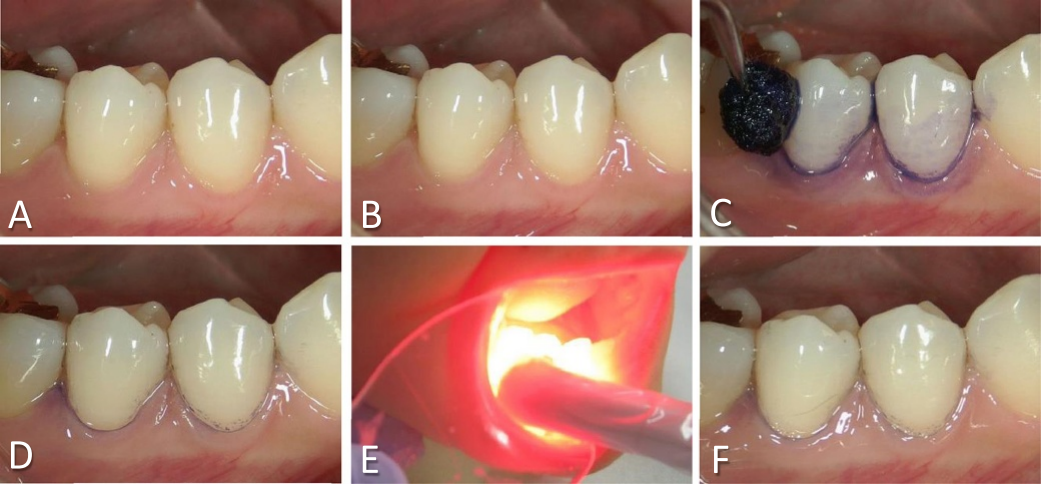
**Photographs showing the aPDT clinical procedure. (A)** Before aPDT. **(B)** After initial removal of the dental plaque (before the first aPDT procedure). **(C)** During the application of TBO. **(D)** After the application of TBO and mouth-rinsing. **(E)** During LED irradiation. **(F)** After LED irradiation.

TBO (1 mg/ml) was gently applied to the buccal and lingual surfaces of the test group teeth using a small cotton pellet. After 10 s, TBO was washed away by rinsing the mouth. After washing, LED was focused at a 90° angle on each buccal and lingual surface of each tooth for 20 s, resulting in a total irradiation time of 80 s on the four surfaces of two premolars in each treatment session (Figure 2). In total, aPDT was applied six times (twice per day, once in the morning, and once in the evening) to the test group teeth for four days. During the trial period, the participants were prohibited from brushing the premolar and the adjacent teeth, and from using mouthwash. On the fourth day, the study concluded and analyses were performed.

#### Determination of the area of dental plaque deposition

After the tooth surfaces were dyed with a plaque-disclosing solution, the buccal and lingual aspects of the first and second premolars in the treatment and control groups were photographed at a 90° angle to the tooth surface. The lingual photographs were taken with a mirror designed especially for mandibular lingual photography (Photographic Mirror ST for Lingual 13262, YDM Corp., Tokyo, Japan). The red color-stained area of dental plaque deposition, as well as the area of the whole tooth surface on the buccal and lingual sides, was determined on the photographs using a software program (Photoshop CS5 Extended; Adobe Systems Inc., CA). To reduce interobserver variability, the determination of the areas was performed independently for each site by two examiners blinded to the experimental groups (A. A. and Y. T.), and the average of the two measurements was used as the representative area value of each site. The percentage of the area of plaque deposition relative to the total tooth surface was calculated.

#### Determination of the number of bacteria in dental plaque collected from the lingual surface of second premolars

The lingual surface of the second premolars was selected as the representative site for plaque collection, because the lingual surface usually has more constant plaque accumulation than the buccal surface, and the second premolar has a larger lingual surface than the first premolar. Supragingival plaque samples were collected with a Gracey curette from the lingual surface of the second premolar prior to treatment (baseline), and 4 days after completion of the clinical trial by blinded examiners (A. A. or Y. T). The number of bacteria was measured using a bacterial counter available at the chair side (Rapid oral bacteria detection apparatus; Panasonic Healthcare, Tokyo, Japan) that was suitable for rapidly counting a large number of bacteria. The counter device employed the dielectrophoretic impedance measurement method, which has been reported to demonstrate a good correlation with the conventional culture method [24].

#### Statistical analysis

Data from the first experiment were evaluated using analysis of variance (ANOVA), followed by Dunnett’s test. The influence of TBO and LED irradiation on fibroblasts was analyzed using two-way factorial ANOVA, followed by Tukey’s test. The comparison of the combined application of 1000 μg/ml TBO and LED irradiation with the two known antiseptics was performed with ANOVA, while the amount of ROS generated during aPDT was analyzed using a *t*-test. Data from the fourth experiment were analyzed using a paired *t*-test. All analyses were performed using the statistical software program JMP 8.0 (SAS Institute, Cary, NC), with the exception of the two-way factorial ANOVA, which was performed by PASW 18.0 (SPSS, IBM, Tokyo, Japan). A *P* < 0.05 was considered statistically significant for all analyses.

## Results

### Experiment 1: bactericidal effect of aPDT on *S. oralis in vitro*

**Figure 3:**
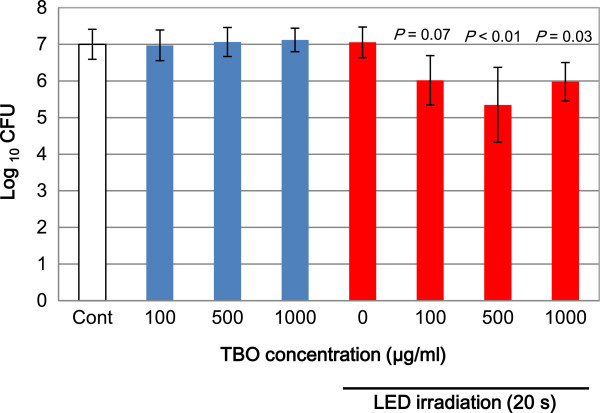
***In vitro*** **bacterial reduction effects of aPDT with TBO and LED on** ***S. oralis*** **.** Blue bars show the effect of TBO only, and red bars present the effect of TBO with LED irradiation. The data represent mean ± SD (n = 5).

In the groups treated with either LED or TBO alone, the numbers of CFUs did not differ from those of the untreated control. Conversely, groups that received TBO + LED exhibited numbers of CFUs that were significantly lower at 500 and 1000 μg/ml (*P* < 0.01 and *P* < 0.05, respectively), compared to the control. The lowest number of CFUs was observed in the group treated with 500 μg/ml TBO + LED (1.42 log reduction, 96.2% killing rate, Figure 3).

### Experiment 2: cytotoxic effect of aPDT on fibroblasts

**Figure 4:**
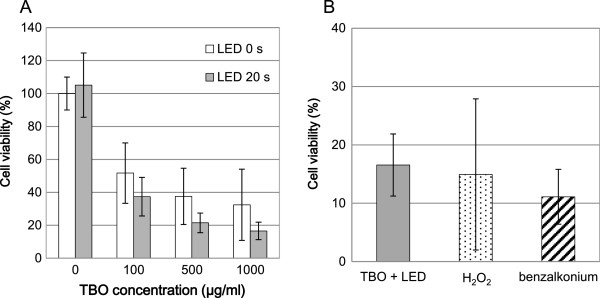
**Cytotoxic effects of aPDT on fibroblasts. (A)** The effect of TBO concentration on cell viability. **(B)** Comparison of cell viability between aPDT and standard antiseptics. The data represent mean ± SD (n = 5).

TBO alone caused a dose-dependent decrease in cell viability. Two-way factorial ANOVA revealed that LED, TBO, and TBO + LED significantly reduced cell viability (*P* < 0.01). Additionally, the application of LED to all concentrations of TBO enhanced the reduction in cell viability (Figure 4A). However, the viability of cells treated with 1000 μg/ml TBO and LED irradiation was not significantly different from the viability of cells treated with 2.5–3% H_2_O_2_ or 0.025% benzalkonium chloride (Figure 4B).

### Experiment 3: analysis of ROSs generated during aPDT

**Figure 5:**
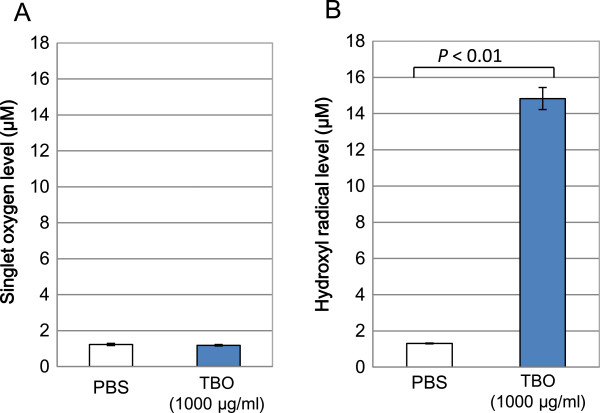
**ROSs detected during the TBO-mediated aPDT procedure. (A)** Singlet oxygen. **(B)** Hydroxyl radical.

**Figure 6:**
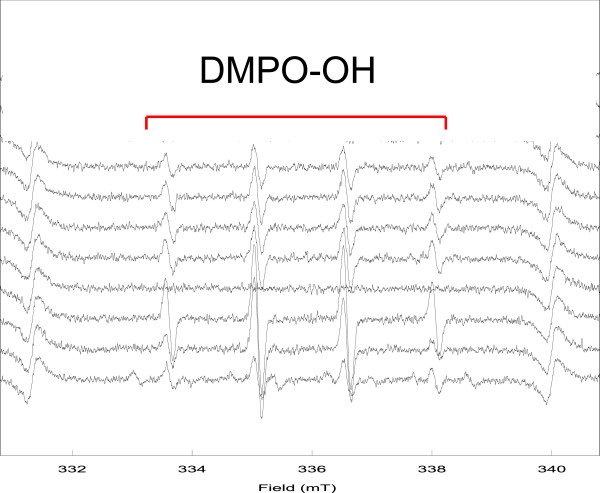
**Typical ESR spectrum during TBO-mediated aPDT procedure.**

No significant differences existed in the production of singlet oxygen between the PBS + LED and the TBO + LED groups (Figure 5A). However, when compared to the control, the production of hydroxyl radical was significantly higher in the TBO + LED group (*P* < 0.01, Figure 5B). The specific DMPO-OH spin adduct, which proves the production of hydroxyl radical, was observed in the TBO + LED group (Figure 6).

### Experiment 4: inhibition effect of aPDT on dental plaque formation

No systemic or local complications were observed after aPDT application during the experimental period. The residual blue staining on the gingiva following TBO application was not visible macroscopically, and was only noted immediately after application. The staining was both minimal and temporary, and caused no esthetic problems for the subjects.

**Figure 7:**
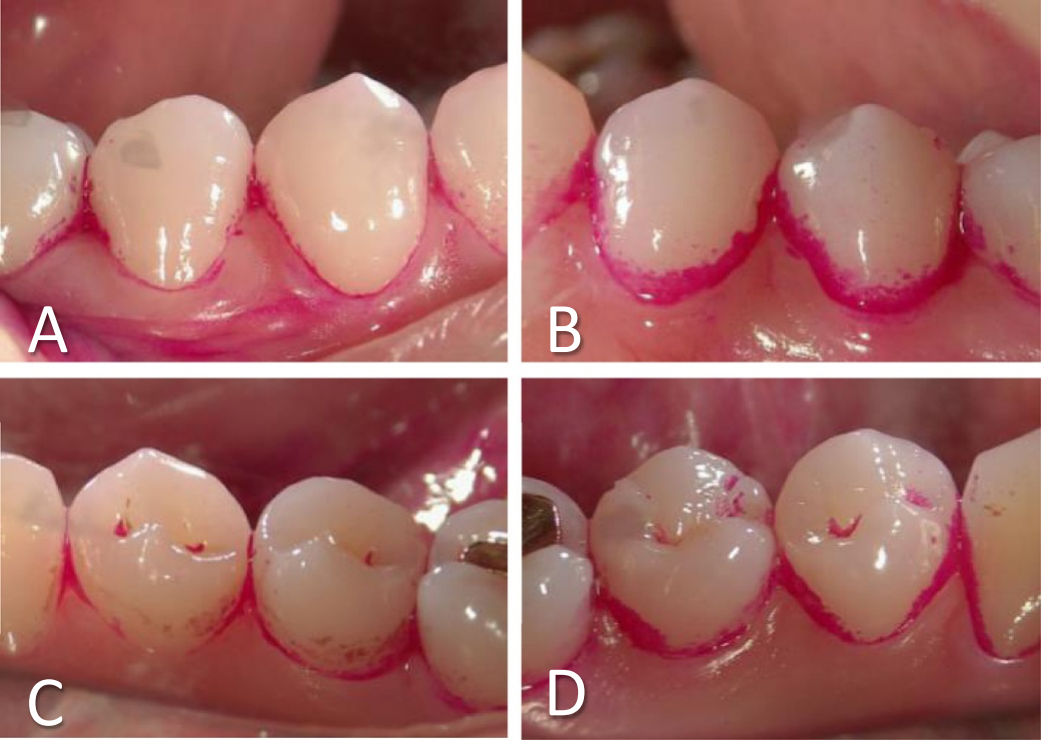
**Results of a clinical trial of the effectiveness of aPDT for inhibition of dental plaque.** Photographs of mandibular premolars of subject #6 after aPDT. Buccal **(A)** or lingual **(C)** surface of aPDT group teeth, and buccal **(B)** or lingual **(D)** surface of control teeth.

**Figure 8:**
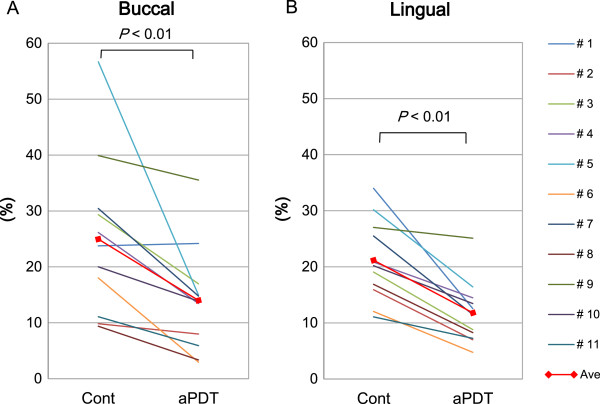
**The ratio of the plaque-deposited area to the total area of buccal (A) and lingual (B) tooth surface in mandibular premolars.**

The plaque formation on the aPDT group teeth was obviously inhibited after day four of aPDT, which was apparent in representative intraoral photographs (Figure 7). The percentages of plaque deposition areas to total buccal (Figure 8A) and lingual tooth surfaces (Figure 8B) were significantly reduced in the aPDT group (buccal = 14 ± 9.1% and lingual = 12 ± 5.4%, mean ± SD, respectively), compared to the control group (buccal = 25 ± 13% and lingual = 21 ± 7.0%, respectively; *P* < 0.01).

**Figure 9:**
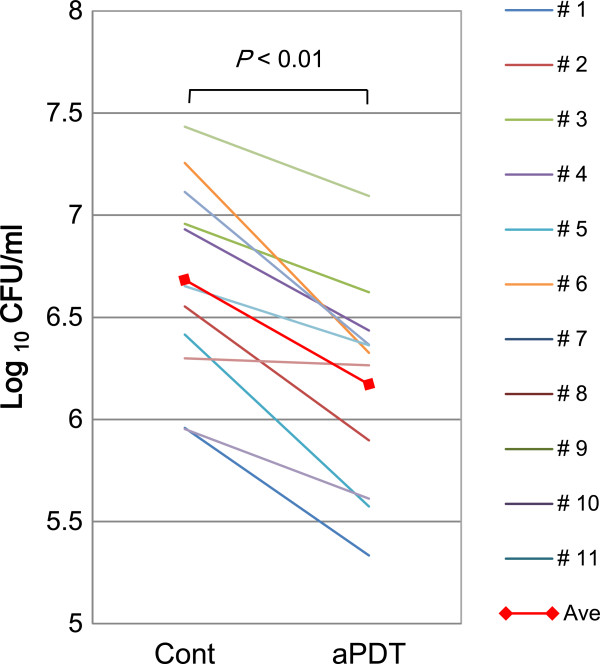
**The number of bacteria in dental plaque collected from the second premolar.**

No significant differences were detected at baseline between the aPDT group (6.17 ± 0.38 log) and the control group (6.29 ± 0.39 log; *P* = 0.49) in the total number of bacteria in the dental plaque collected from the lingual surface of the second premolar. However, the total number of bacteria in the dental plaque was significantly lower 4 days after the clinical trial in the aPDT group (6.17 ± 0.49 log) compared to the control group (6.68 ± 0.48 log; *P* < 0.01; Figure 9).

## Discussion

To our knowledge, this is the first clinical trial to demonstrate the effectiveness of aPDT in inhibiting dental plaque formation on teeth without inducing harmful effects to host tissues. Further, this study confirmed that TBO with red LED effectively reduced the bacteria *S. oralis,* which is known as an initial colonizer in dental plaque formation, and is often detected in the blood of patients who suffer from infectious endocarditis [25, 26]. The use of TBO with a high-power LED device and a 20 s irradiation time, led to a significant dose-dependent reduction in CFUs *in vitro*. However, the reduction was less when 1000 μg/ml TBO (final concentration of 500 μg/ml) was used than when 500 μg/ml (final concentration of 250 μg/ml) was applied. A possible explanation was the fact that the blue-colored 1000 μg/ml TBO mixed bacterial solution was too dark for the red light to penetrate through the solution to the bottom of the 96-well plate, and thus the sensitization of TBO by LED irradiation was potentially blocked. Nonetheless, 1000 μg/ml TBO was used because in clinical situations, the immediate dilution of TBO with saliva and its superficial spread on the tooth surface could lead to the more efficient light sensitization of TBO than witnessed *in vitro*. In the present study, the focus was only on *S. oralis*; however, the formation of dental biofilm consists of different primary colonizers and complex inter-microbial interactions. Therefore, several other plaque forming bacteria including *Actinomyces viscosus* and *Streptococcus sanguis* should be investigated in future studies.

With respect to the safety of the procedure, TBO alone negatively influenced the viability of fibroblasts in a concentration-dependent manner, and the application of LED enhanced the affect. However, the *in vitro* reduction in cell viability under the present aPDT conditions (1000 μg/ml TBO with 20 s LED irradiation) did not exceed that observed with other antiseptics, which indicated that the cytotoxicity of aPDT was within the conventional levels. Additionally, although prior reports have indicated the resistance of bacteria to antiseptics such as benzalkonium chloride, which is a quaternary ammonium cationic surfactant that interrupts the lipid membrane of cells [27, 28], the lack of bacterial resistance following application of aPDT would be another benefit in clinical application [29]. In the present study, however, we only observed acute cytotoxicity at 2 h after single application of aPDT, and thus further studies are required to investigate the long-term influence of aPDT on cell proliferation, as well as the cumulative action following repeated applications.

The analysis of ROS generated during TBO-mediated aPDT resulted in the novel finding that the hydroxyl radical was the primary product. Although the mechanism of aPDT [30] is generally thought to take place by a Type I process, which produces a hydroxyl radical by electron transfer, or a Type II process, which yields singlet oxygen by energy transfer, no modality of cell death by TBO-mediated aPDT has been clarified. Singlet oxygen is regarded as the major damaging species in aPDT [31], and the common photosensitizer methylene blue is a known producer of singlet oxygen. In our previous pilot study (data not shown), we confirmed the production of singlet oxygen in methylene blue-mediated aPDT. However, ESR analysis clearly revealed that the hydroxyl radical was the predominant product of TBO-mediated aPDT. Further, Type I machinery was speculated to play an important role in this aPDT procedure.

Following the results of the *in vitro* experiments, we attempted the clinical application of aPDT for the inhibition of dental plaque formation, and observed a significant suppression of plaque deposition on teeth treated with aPDT. The four day duration of the clinical trial was short, and thus a longer duration was desirable. However, the duration of this pilot study was limited to four days, due to consideration of the physical and mental stresses caused by prohibiting tooth brushing, and the concerns of volunteer dentists regarding the occurrence of tooth decay and gingival inflammation.

Regarding the mechanism of aPDT, a recent proteomic approach by Dosselli *et al*. [32] revealed that aPDT delays the growth of bacteria, and reduces the capacity of bacteria for glucose consumption. Combined, the bacterial killing effect and the retardation of bacterial growth with aPDT could reduce plaque deposition on teeth.

Nonetheless, the negative aspects of the clinical use of aPDT should be considered. Some reports have noted tooth staining with TBO [33], however, residual staining of teeth and gingival tissue with TBO after the aPDT procedure was not visible, and did not present a problem in this clinical trial. Additionally, light energy has a biological effect on the activations of cells and tissues. Therefore, various positive and negative effects of the irradiation of teeth and gingival tissues, such as reducing gingival inflammation or inducing the calcification of dental pulp [34], need to be clarified. In particular, although the performance of aPDT was only short term in this present study, more attention to the cumulative action of potential side effects should be paid in the long-term repeated usage for daily plaque control.

Additionally, in this clinical trial, the reduction rate in the plaque-deposition area was on average no more than approximately 56% following aPDT treatments, probably because the susceptibility of bacteria to aPDT was thought to be much lower in biofilms than in the planktonic condition [35]. Hence, modification of the system for delivery of the photosensitizer into the biofilm should be examined for the effective enhancement of the inhibitory effects of aPDT on dental plaque formation. An example of a possible modification is the use of an antibody conjugated to photosensitizers [36]. Similarly, the optimal power and time of LED irradiation, in combination with the frequency of aPDT procedure, should be clarified. In addition, the present pilot study was limited by the employment of healthy volunteer dentists as subjects, as well as by the small number of participants and the short duration of the clinical trial. Consequently, further investigations involving suitable conditions for clinical plaque control in a larger number of subjects and for a longer trial duration are required to confirm the safe and effective aPDT procedure compared with conventional treatments. In the future, aPDT could be used for plaque control at an office equipped with a specific appliance, or at home with the combination of tooth paste containing a photosensitizer and a light-emitting toothbrush [5].

## Conclusions

The application of aPDT significantly suppressed the formation of dental plaque, which indicated that aPDT may be a promising alternative or adjunct method to mechanical means in dental plaque control for oral care. This study provides initial data on a potentially new approach, and thus further validation to determine optimum conditions including dye concentration, the modification of dye application, the light power, and the frequency of the procedure, as well as any potential adverse effects, are required in order to establish a safe and effective aPDT procedure.

## Electronic supplementary material

Additional file 1: **CONSORT checklist items.** (ZIP 12 KB)
